# Mechanical Ventilation and the Titer of Antibodies as Risk Factors for the Development of Transfusion-Related Lung Injury 

**DOI:** 10.1155/2012/720950

**Published:** 2012-06-04

**Authors:** A. P. J. Vlaar, M. T. Kuipers, J. J. Hofstra, E. K. Wolthuis, C. W. Wieland, J. J. T. H. Roelofs, L. Boon, M. J. Schultz, R. Lutter, N. P. Juffermans

**Affiliations:** ^1^Department of Intensive Care Medicine, Academic Medical Center, 1105 AZ Amsterdam, The Netherlands; ^2^Laboratory of Experimental Intensive Care and Anesthesiology (LEICA), Academic Medical Center, 1105 AZ Amsterdam, The Netherlands; ^3^Department of Anesthesiology, Academic Medical Center, 1105 AZ Amsterdam, The Netherlands; ^4^Department of Pathology, Academic Medical Center, 1105 AZ Amsterdam, The Netherlands; ^5^Bioceros B.V., 3584 CM Utrecht, The Netherlands; ^6^Departments of Respiratory Medicine and Experimental Immunology, Academic Medical Center, 1105 AZ Amsterdam, The Netherlands

## Abstract

*Purpose*. Onset of transfusion-related acute lung injury (TRALI) is suggested to be a threshold-event. Data is lacking on the relation between titer of antibodies infused and onset of TRALI. We determined whether onset of TRALI is dependent on the titer of MHC-I antibodies infused in a combined model of ventilator-induced lung injury and antibody-induced TRALl. *Methods*. BALB/c mice were ventilated for five hours with low (7.5 ml/kg) or high (15 ml/kg) tidal volume. After three hours of MV, TRALI was induced by infusion of 0.5 mg/kg, 2.0 mg/kg or 4.5 mg/kg MHC-I antibodies. Control animals received vehicle. After five hours of MV, animals were sacrificed. *Results*. MV with high tidal volumes resulted in increased levels of all markers of lung injury compared to animals ventilated with low tidal MV. In ventilator-induced lung injury, infusion of 4.5 mg/kg of antibodies further increased pulmonary wet-to-dry ratio, pulmonary neutrophil influx and pulmonary KC levels, whereas infusion of lower dose of antibodies did not augment lung injury. In contrast, mice ventilated with low tidal volumes did not develop lung injury, irrespective of the dose of antibody used. *Conclusions*. In the presence of injurious MV, onset of TRALI depends on the titer of antibodies infused.

## 1. Introduction

Transfusion-related acute lung injury (TRALI) is the leading cause of transfusion-related morbidity and mortality [1–3]. The pathogenesis of TRALI has not been fully elucidated. A “two hit” event has been postulated [4]. The first event is an inflammatory condition of the patient (e.g., sepsis, recent surgery) causing sequestration and priming of neutrophils in the pulmonary compartment. The second event is the transfusion, containing either antibodies or bioactive lipids that have accumulated during blood storage, stimulating the primed neutrophils to release proteases. The result is endothelial damage, capillary leak, and extravasation of neutrophils, finally resulting in pulmonary edema, that is, TRALI.

The association between HLA antibodies in donor plasma and TRALI is not always apparent [5–7]. A threshold model has been suggested [8], in which a threshold must be overcome to induce a TRALI reaction. Factors that determine the threshold are the predisposition of the patient that determines priming of the lung neutrophils and the ability of the mediators in the transfusion to cause activation of primed neutrophils. A strong antibody-mediated response can cause severe TRALI in an otherwise “healthy” recipient. When activation status is too low, it is possible that priming factors in the transfusion are not strong enough to overcome the threshold. The threshold model may explain why the estimated incidence of TRALI is higher in cardiac surgery and critically ill patients [9–12]. These patients often suffer from an inflammatory condition, which may contribute to neutrophil priming, after which transfusion of mediators with low activating capacity may be sufficient to overcome the threshold to induce a TRALI reaction [11]. Indeed, LPS priming allowed for lower amounts of antibody needed to induce TRALI [13]. In line with this, sepsis was found to be a risk factor for TRALI in critically ill patients [9, 11]. In addition, we recently showed that mechanical ventilation (MV) aggravates the course of a TRALI reaction in a murine model [14]. Although data on patient-related risk factors is increasing [9–11, 15], data on the threshold titer of antibodies needed to induce TRALI is sparse.

Blood transfusion significantly contributes to morbidity and mortality in trauma, general surgery, and critically ill patients [16]. Understanding the interaction between the underlying condition of the patient and the concentration of antibodies needed to cause TRALI may help define interventions that diminish the risk of TRALI. To investigate whether onset of TRALI depends on the titer of MHC-I antibodies infused, we tested three concentrations of MHC-I antibodies in a combined model of TRALI and ventilator-induced lung injury [17, 18].

## 2. Materials and Methods

The study was approved by the Animal Care And Use Committee of the Academic Medical Center at the University of Amsterdam, Amsterdam, The Netherlands. Animal procedures were carried out in compliance with the Institutional Standards for Human Care and Use of Laboratory Animals.

### 2.1. MHC I mAb Production

 A hybridoma (34-1-2S) was purchased from the American Type Culture Collection that produces a mAb against H2K^d^ (IgG_2a_, *κ*). The hybridoma was grown in tissue culture medium containing 1% fetal bovine serum and incubated at 37°C and 5% CO_2_. Hybridoma supernatant was collected and filtered through a 0.2 *μ*m filter. The MHC I mAb was purified using protein A Sepharose affinity chromatography and dialyzed overnight in PBS (pH 7.4). The protein concentration of the mAb was spectrophotometrically determined using Bio-Rad protein reagent. The mAb stock solution (2.0–2.5 mg/mL) was frozen at −80°C until the time of the experiments.

### 2.2. Mice

Experiments were performed with healthy male BALB/c mice (total *n* = 96, *n* = 12 per group, Charles River, Someren, the Netherlands), aged 8 to 10 weeks, with weights ranging from 19 to 25 g. Animals were mechanically ventilated with two different MV strategies for 5 hours and received 0.5, 2.0, or 4.5 mg/kg MHC-I class antibodies infusion after 3 hours of ventilation. In a previous study using the same model, no differences between markers of lung injury were found between animals receiving ISO-type antibody and animals receiving vehicle infusion [14]. Therefore, only controls receiving vehicle infusion were used.

### 2.3. Instrumentation and Anesthesia

Anesthesia was achieved with intraperitoneal injection (i.p.) of a mix of ketamine (Eurovet Animal Health B.V., Bladel, The Netherlands), medetomidine (Pfizer Animal Health B.V., Capelle a/d IJssel, The Netherlands), and atropine (Pharmachemie, Haarlem, the Netherlands) (KMA). Induction anesthesia consisted of injection of KMA “induction”—mix: 7.5 *μ*L per gram of body weight of 1.26 mL 100 mg/mL ketamine, 0.2 mL 1 mg/mL medetomidine, and 1 mL 0.5 mg/mL atropine in 5 mL normal saline. Throughout the experiments rectal temperature was maintained between 36.0 and 37.5°C using a warming path. Maintenance anesthesia consisted of 10 *μ*L per gram body weight of a mix of 0.72 mL 100 mg/mL ketamine, 0.08 mL 1 mg/mL medetomidine, and 0.3 mL 0.5 mg/mL atropine in 20 mL normal saline administered via an intraperitoneal catheter (PE 10 tubing, BD, Breda, The Netherlands) every hour.

### 2.4. Mechanical Ventilation Strategies

A Y-tube connector, 1.0 mm outer diameter and 0.6 mm inner diameter (VBM Medizintechnik GmbH, Sulz am Neckar, Germany), was surgically inserted into the trachea under general anesthesia. Mice were placed in a supine position and connected to a ventilator (Servo 900 C, Siemens, Sweden). Mice were pressure controlled ventilated with either an inspiratory pressure of 10 cm H_2_O (resulting in lung-protective V_T_ ~ 7.5 mL/kg; low V_T_, LV_T_)  *or* an inspiratory pressure of 18 cm H_2_O (resulting in injurious V_T_ ~ 15 mL/kg; high V_T_, HV_T_). Respiratory rate was set at 110 breaths/min and 50 breaths/min with LV_T_ and HV_T_, respectively. These respiratory settings resulted in normal PaCO_2_ values after 5 h of MV [18]. PEEP was set at 2 cm H_2_O with both MV strategies. The fraction of inspired oxygen was kept at 0.5, and inspiration to expiration ratio was set at 1 : 1. A sigh (sustained inflation with 30 cm H_2_O) for 5 breaths was performed every 30 minutes. Mice received an intraperitoneal bolus of 1 mL normal saline 1 hour before start of anesthesia and initiation of MV, followed by 0.2 mL sodium carbonate (200 mmol/L NaHCO_3_) administered via the intraperitoneal catheter every 30 minutes until the end of MV. After 3 hours of MV, the jugular vein was isolated. Using a 30-gauge sterile needle attached to PE-10 tubing, venous blood was aspirated from the jugular vein to verify intravascular placement of the needle and to remove a sample of blood (~200 *μ*L). Mice were given an i.v. volume-matched injection (150–250 *μ*L) of either 0.5, 2.0, or 4.5 mg/kg MHC-I class antibodies. Controls received vehicle. The skin was sutured with 6-0 silk suture, and the mice were sacrificed after two more hours of MV.

### 2.5. Hemodynamic Monitoring

Systolic blood pressure and heart rate were noninvasively monitored using a murine tail-cuff system (AD Instruments, Spechbach, Germany). The data were recorded on a data acquisition system (PowerLab/4SP, AD Instruments). Systolic blood pressure and heart rate were averaged from three consecutive measurements.

### 2.6. Study Groups and Sampling

At the end of the experiment animals were sacrificed, and bronchoalveolar lavage fluid (BALF) was obtained from the right lung (*n* = 6), by instilling three times 0.5 mL aliquots of saline by a 22-gauge Abbocath-T catheter (Abbott, Sligo, Ireland) into the trachea. Approximately, 1.0 mL of lavage fluid was retrieved per mouse, and cell counts were determined using a hemocytometer (Beckman Coulter, Fullerton, CA, USA). Differential counts were done (up to 100 cells per slide) on cytospin preparations stained with a modified Giemsa stain, Diff-Quick (Dade Behring AG, Düdingen, Switzerland). Supernatant was stored at −80°C for cytokine measurement. The left lung was weighed and dried for three days in an oven at 65°C. The ratio of wet weight to dry weight represents tissue edema. Another 6 mice were used for blood gas analysis from blood sampled from the carotid artery. The lungs of these mice were fixed in 4% formalin and embedded in paraffin for histopathology. 4 *μ*m sections were stained with hematoxylin-eosin (H&E) and analyzed by a pathologist who was blinded for group identity. To score lung injury, we used a modified VILI histology scoring system as previously described [19]. In short, four pathologic parameters were scored on a scale of 0–4: (a) alveolar congestion, (b) hemorrhage, (c) leukocyte infiltration, and (d) thickness of alveolar wall/hyaline membranes. A score of 0 represents normal lungs; 1, mild, <25% lung involvement; 2, moderate, 25–50% lung involvement; 3, severe, 50–75% lung involvement; 4, very severe, >75% lung involvement. The total histology score was expressed as the sum of the score for all parameters.

### 2.7. Assays

Cytokine and chemokine levels in the BALF were measured by enzyme-linked immunosorbent assay (ELISA) according to the manufacturer's instructions. Interleukin (IL)-6, macrophage inflammatory protein (MIP)-2 and keratinocyte-derived chemokine (KC) assays were all obtained from the R&D Systems (Abingdon, UK).

### 2.8. Statistical Analysis

All data in the results are expressed as means ± SEM. To detect differences between groups, the Dunnett method was used, in conjunction with two-way analysis of variance and the Mann Whitney *U* test. A *P* value of <0.05 was considered significant. All statistical analyses were carried out using SPSS 12.0.2 (SPSS, Chicago, IL).

## 3. Results

### 3.1. Hemodynamic and Ventilatory Monitoring

 All ventilated animals survived the 5 hours of MV. Blood gas analysis revealed adequate gas exchange as previously shown [14]. Arterial pressure and heart rate remained stable in all animals throughout the experiment, with no differences noted between before and after infusion, nor between groups (data not shown).

### 3.2. Effect of Mechanical Ventilation with High Tidal Volume

**Figure 1:**
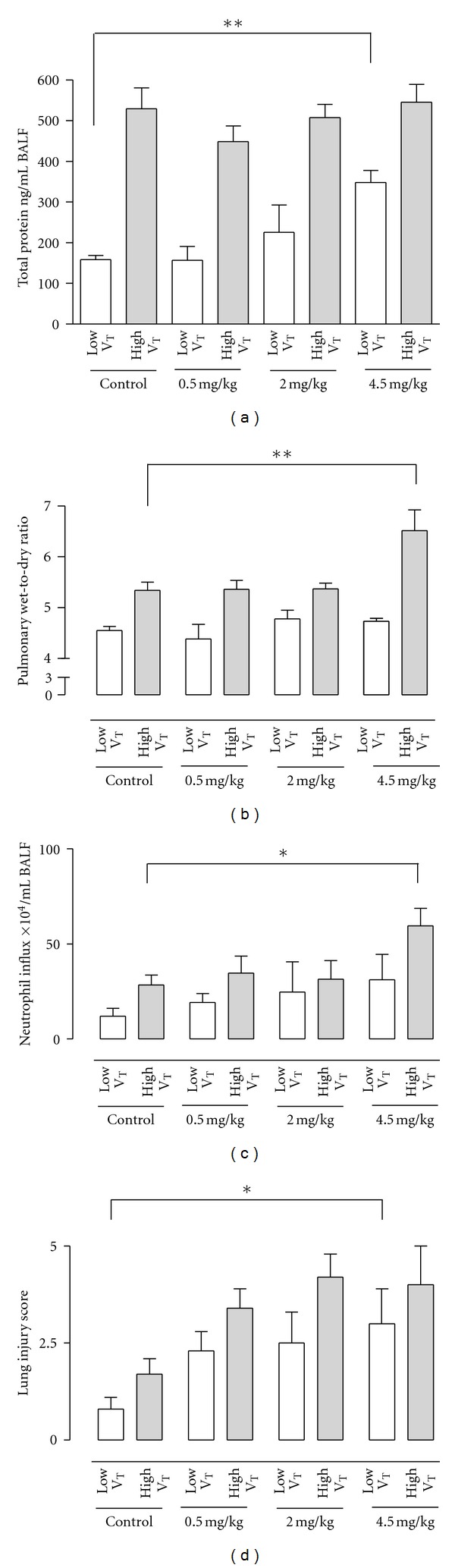
(a) Total protein in the bronchoalveolar lavage fluid (BALF) (*n* = 12 per group), (b) wet-to-dry ratio of the lungs (*n* = 6 per group), (c) neutrophil influx in bronchoalveolar lavage fluid (*n* = 12 per group), and (d) histopathological examination of lung tissue (*n* = 6 per group). Low V_T_ = mice ventilated with a tidal volume of 7.5 mL/kg; high V_T_ = mice ventilated with a tidal volume of 15 mL/kg. Dose of MHC-I antibodies infused 0.5, 2.0, and 4.5 mg/kg. Controls received vehicle. BALF = bronchoalveolar lavage fluid. *P < 0.05, **P < 0.01.

**Figure 2:**
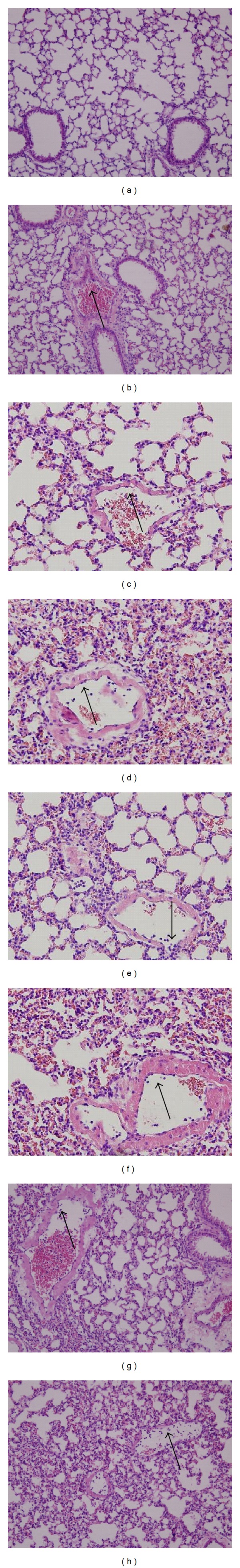
Histologic sections of hematoxylin-and-eosin-stained mice lungs at 100x magnification after mechanical ventilated using low ventilation tidal (V_T_) (7.5 mL/kg) or high V_T_ (15 mL/kg). Ventilated animals receiving 0.5 mg/kg, 2.0 mg/kg or 4.5 mg/kg MHC-I antibody (*n* = 6 per group). (a) low V_T_ control, (b) high V_T_ control, (c) 0.5 mg/kg low V_T_, (d) 0.5 mg/kg high V_T_, (e) 2.0 mg/kg low V_T_, (f) 2.0 mg/kg high V_T_, (g) 4.5 mg/kg low V_T_, (h) 4.5 mg/kg high V_T_. Neutrophils sequestrated in the vasculature (arrow; (a)–(h)). Increased pulmonary edema and neutrophil extravasation ((d), (f)–(h)).

**Figure 3:**
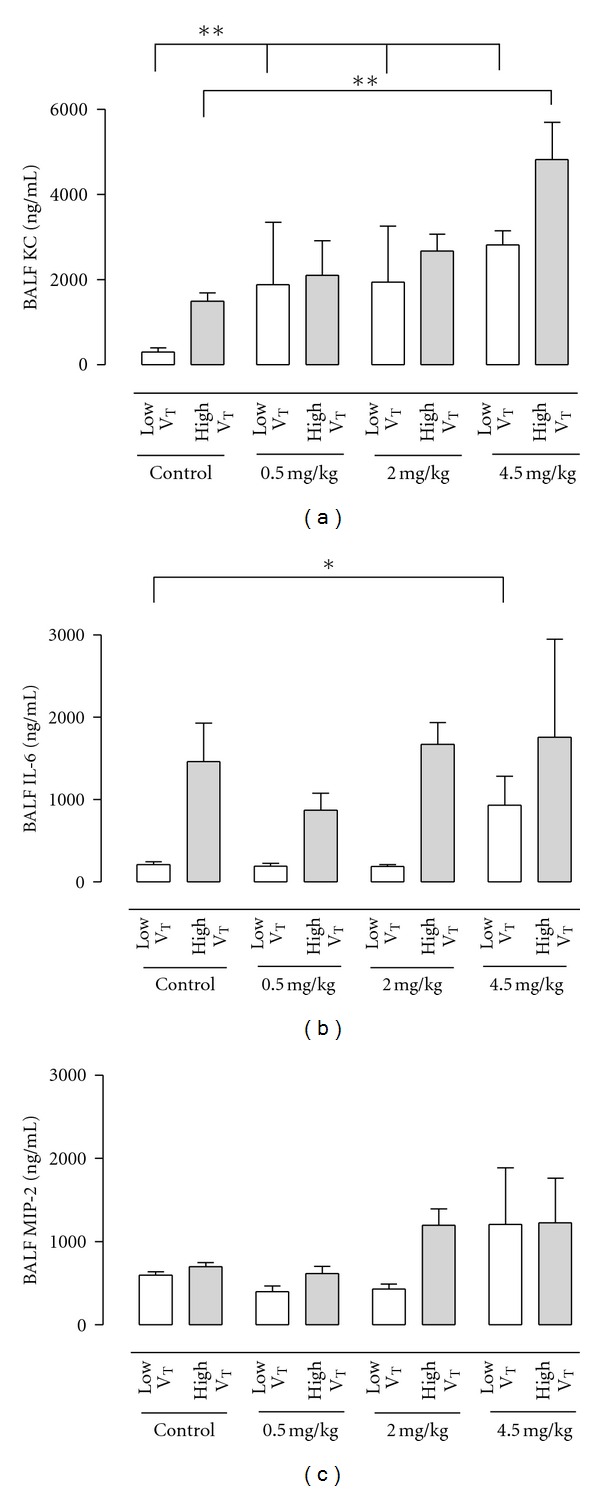
(a) Keratinocyte-derived chemokine (KC) (*n* = 12 per group), (b) interleukin (IL)-6 (*n* = 6 per group) and (c) MIP-2 concentrations (*n* = 6 per group) in the bronchoalveolar lavage fluid (BALF). Ventilated animals receiving 0.5 mg/kg, 2.0 mg/kg, or 4.5 mg/kg MHC-I antibody. Animals were low V_T_ (tidal of 7.5 mL/kg) and high V_T_ (tidal of 15 mL/kg). Controls received vehicle. *P < 0.05, **P < 0.01.

Animals receiving high tidal MV showed pulmonary neutrophil sequestration with an increased wet-to-dry ratio of the lungs and increased pulmonary protein leakage compared to animals receiving low tidal MV (Figures 1 and 2, *P* < 0.01), consistent with previous results [14, 18, 20]. Also, high tidal MV resulted in increased pulmonary levels of KC and IL-6 compared to low tidal MV (Figures 3(a) and 3(b), *P* < 0.01).

### 3.3. Effect of MHC-I Antibody Infusion in Mice Ventilated with Injurious Tidal Volumes

The increased wet-to-dry ratio of the lungs induced by high tidal MV was further increased after infusion with a high dose of MHC-I antibodies (4.5 mg/kg) compared to animals receiving vehicle (Figure 1(b), *P* < 0.01). Animals ventilated with high tidal MV and receiving high dose of MHC-I antibodies had increased neutrophil influx in the BALF and increased pulmonary levels of KC compared to animals receiving vehicle (Figures 1(c) and Figure 3, *P* < 0.05 and *P* < 0.01, resp.). This effect was also seen for pulmonary levels of IL-6 and MIP-2, although not reaching statistical significance (Figures 3(b) and 3(c), ns). No difference was seen in total protein leakage in the BALF between animals receiving MHC-I antibodies and controls (Figure 1(a), ns). In animals ventilated with injurious ventilator settings, a low dose (0.5 mg/kg) of MHC-I antibodies induced a nonsignificant trend for pulmonary neutrophil sequestration, increase of pulmonary levels of KC, and increase in lung injury score compared to animals receiving vehicle (Figures 1, 2, and 3).

### 3.4. Effect of MHC-I Antibody Infusion in Mice Ventilated with Protective Tidal Volumes

In animals ventilated with low tidal volume, infusion of MHC-I antibodies did not increase wet-to-dry ratio or neutrophil influx, irrespective of the concentration used (Figure 1). Infusion of high dose (4.5 mg/kg) MHC-I antibodies in animals ventilated with protective ventilator settings resulted in an increase in total protein leakage in the BALF, lung injury score and pulmonary KC, and IL-6 levels compared to animals receiving vehicle (Figures 1 and 3).

## 4. Discussion

Here, we describe the effect of different doses of antibodies in inducing TRALI in a clinically relevant model of mechanically ventilated animals. We hypothesized that in, the presence of injurious MV (“first hit”), lower titers of antibodies would result in aggravation of lung injury, which would not occur in the presence of “protective” MV. We found evidence for a titer threshold in the induction of TRALI for some lung injury parameters, suggesting that onset of TRALI depends both on the “first hit” as well as on the amount of MHC-I antibodies infused. However, this effect was not ubiquitous for all parameters of lung injury. Also, we found that already low concentrations of MHC-I antibodies can induce lung injury, even in the presence of a “protective” MV strategy.

Originally, TRALI was thought to be a “single hit” antibody-mediated reaction, in which antibodies in the blood product react with a matching antigen in the recipient, leading to pulmonary edema [21]. Then, evidence emerged that host factors play an important role in determining whether or not a TRALI reaction occurs [9, 11, 12]. The concept that the presence of a “first hit” may be required for a TRALI reaction has been shown in several non-immune-mediated TRALI models. Infusion of stored blood products or bioactive lipids requires LPS priming before inducing a TRALI reaction [22–26]. Recent studies show that the “two-event model” also holds true for immune-mediated TRALI using LPS as a “first hit” [13, 22]. Here, we confirm the finding of a threshold dependency with a clinically relevant “first hit” of injurious MV for some parameters of TRALI, the most apparent being the increase in pulmonary edema, an effect that was not observed after infusion of lower concentrations of MHC-I antibodies. However, not all parameters of lung injury showed a similar threshold, including protein leakage. An explanation for this finding may be that injurious MV already elicits protein leakage [18], as also seen in this study, which could not be further enhanced by MHC-I antibodies.

Our findings may suggest that mechanically ventilated patients may be at risk for the onset of TRALI. This accords with the finding that mechanically ventilated critically ill patients are at risk for onset of ALI after transfusion with fresh frozen plasma [27]. The mechanism of the synergistic effect of injurious MV and TRALI [14] may be recruitment of neutrophils to the pulmonary compartment induced by MV [28], resulting in a proinflammatory response (i.e., priming), resembling a “first hit” in TRALI models. The primed pulmonary neutrophils may be prone to activation after a “second hit,” which results in TRALI. In the absence of the primed neutrophils (i.e., “first hit”), the “second hit” may not overcome the threshold and TRALI will not occur [22]. In support of this view, resting neutrophils express HLA class II antigens at very low levels, whereas cytokine-activated neutrophils express increased HLA class II antigens [29, 30]. In line with the thought that MV may serve as a predisposing clinical condition in the onset of TRALI, we found that lung injury score worsened already after infusion of the lowest dose of MHC-I antibodies, irrespective of the ventilation strategy. Although it is not apparent from this study whether MV can prime pulmonary neutrophils, thereby increasing susceptibility for a TRALI reaction, we conclude that already low dose of MHC-I antibodies elicits pulmonary inflammation in the presence of MV. Whether or not MV may account for the high incidence of TRALI among the critically ill remains to be determined [9–11].

The finding that injurious MV settings can facilitate the onset and course of a TRALI reaction, allowing for a lower titer of MHC-I antibodies to induce TRALI, may have relevance for the prevention of TRALI. Recent clinical data indicate that the volume of cognate antibodies transfused is indeed a risk factor for TRALI [31]. Plasma volumes in red blood cell units as small as 10–20 mL or a single buffy coat donor from a pooled platelet product was reported sufficient to cause TRALI [32]. Plasma from multiparous donors contains higher levels of leukocyte and/or neutrophil antibodies due to sensitization during labour compared to male donors. Excluding female donors for high-volume plasma components in the UK and The Netherlands reduced the onset of TRALI in surgical and critically ill patient populations [33–35]. However, deferring women from plasma donation has a strong impact on blood supply. Further research is needed to establish which titer of antibodies can be safely transfused and whether this concentration threshold differs between relatively healthy recipients and mechanically ventilated patients undergoing surgery or suffering from an inflammatory condition.

There are limitations to this study which hamper the relevance of our findings for clinical practice. Obviously, results of an animal model cannot be translated directly to the clinical setting. We do not know whether 4.5 mg/kg MHC-I is a clinical relevant dose. Also, we infused a component of the blood product which does not reflect the clinical situation. Although MHC-I antibody is the main cause of antibody mediated TRALI, we cannot rule out that other blood components may play a role in the onset of lung injury. Results may, however, serve to explore the hypothesis that mechanically ventilated patients benefit from a policy of plasma with a reduced titer of antibodies.

## 5. Conclusion

In the presence of injurious MV, onset of TRALI is dependent on the amount of MHC-I antibodies infused, supporting the threshold model. Results may suggest that decreasing the concentration of antibodies in blood products may lower the risk of a TRALI reaction in mechanically ventilated patients.
